# Inhibition of Human Kallikrein 5 Protease by Triterpenoids from Natural Sources

**DOI:** 10.3390/molecules22111829

**Published:** 2017-10-27

**Authors:** Yosuke Matsubara, Takashi Matsumoto, Junichi Koseki, Atsushi Kaneko, Setsuya Aiba, Kenshi Yamasaki

**Affiliations:** 1 Tsumura Research Laboratories, Tsumura & Co., Ibaraki 300-1192, Japan; matsumoto_takashi@mail.tsumura.co.jp (T.M.); htkj9626m@mineo.jp (J.K.); kaneko_atsushi@mail.tsumura.co.jp (A.K.); 2 Department of Dermatology, Tohoku University Graduate School of Medicine, Miyagi 980-8574, Japan; saiba@med.tohoku.ac.jp (S.A.); kyamasaki@med.tohoku.ac.jp (K.Y.)

**Keywords:** kallikrein 5, kallikrein 7, triterpenoid, ursolic acid, tumulosic acid, Jumihaidokuto, skin barrier, cathelicidin, LL-37

## Abstract

Stratum corneum tryptic enzyme kallikrein 5 (KLK5) is a serine protease that is involved in the cell renewal and maintenance of the skin barrier function. The excessive activation of KLK5 causes an exacerbation of dermatoses, such as rosacea and atopic dermatitis. Some triterpenoids are reported to suppress the serine proteases. We aimed to investigate whether bioactive triterpenoids modulate the KLK5 protease. Nineteen triterpenoids occurring in medicinal crude drugs were evaluated using an enzymatic assay to measure the anti-KLK5 activity. The KLK5-dependent cathelicidin peptide LL-37 production in human keratinocytes was examined using immunoprecipitation and Western blotting. Screening assays for evaluating the anti-KLK5 activity revealed that ursolic acid, oleanolic acid, saikosaponin b_1_, tumulosic acid and pachymic acid suppressed the KLK5 protease activity, although critical molecular moieties contributing to anti-KLK5 activity were unclarified. Ursolic acid and tumulosic acid suppressed the proteolytic processing of LL-37 in keratinocytes at ≤10 μM; no cytotoxicity was observed. Both triterpenoids were detected in the plasma of rats administered orally with triterpenoid-rich crude drug Jumihaidokuto. Our study reveals that triterpenoids, such as ursolic acid and tumulosic acid, modulate the KLK5 protease activity and cathelicidin peptide production. Triterpenoids may affect the skin barrier function via the regulation of proteases.

## 1. Introduction

Stratum corneum tryptic enzyme kallikrein 5 (KLK5) is a serine protease that is expressed in the epidermis and is involved in the cell renewal and maintenance of skin barrier function. KLK5 plays a central role in the degradation of corneodesmosome, which is the main adhesive structure in the cornified cell layer. KLK5 is also involved in the activation of other epithelial serine proteases such as kallikrein- and matriptase-family protease [1,2]. Although KLK5 is important for the maintenance of skin homeostasis, its overactivation can impair the skin barrier function and contribute to the development of various dermatoses. Lympho-epithelial Kazal-type inhibitor 1 (LEKTI-1), an endogenous inhibitor of serine protease, was shown to regulate KLK5 and the family proteases in healthy humans [3,4]. In Netherton syndrome, which is caused by loss-of-function mutations in SPINK5 encoding for LEKTI, an aberrant KLK5 protease activity in patients with this syndrome causes extensive skin desquamation, inflammation, allergic manifestations, and hair shaft defects [4]. Cathelicidin antimicrobial peptides (CAMPs) are cleaved into the active antimicrobial peptide LL-37 by both KLK5 and kallikrein 7 (KLK7) [5]. Patients with rosacea have elevated levels of cathelicidin and KLK5 protease; an excessive production of LL-37 is suspected to contribute the trigger and the exacerbation of rosacea [6,7,8]. Moreover, the overactivation of KLK5 induces proinflammatory cytokines, such as TSLP and TNF-α, which are implicated in the development of dermatoses such as pruritus and atopic dermatitis [9,10]. These findings indicate that KLK5 is the master protease that is closely involved in the pathogenesis as well as exacerbation of refractory dermatoses. Because there is no drug or therapy to control KLK5 so far, the development of a therapeutic drug and/or strategy for the control of skin serine proteases is desired [11].

Several studies have shown that some triterpenoids are bioactive natural products that suppress the serine protease activity. Plant-derived pentacyclic tritepenoids, such as oleanolic acid, ursolic acid, and β-boswellic acid have been reported to inhibit the esterase activity in human neutrophils [12,13]. Some triterpenoids isolated from the Compositae flower were shown to suppress trypsin and chymotrypsin activities; an intramolecular moiety has been discussed to be essential for their activities [14]. In addition, the beneficial effects of various triterpenoids have been demonstrated in animal models of dermatitis and skin barrier function disruption [15,16,17]. To the best of our knowledge, the direct interaction between triterpenoids and KLK enzymes has not yet been reported.

To address whether bioactive triterpenoids have anti-KLK5 activity, we employed a pharmaceutical-grade traditional Japanese medicine, Jumihaidokuto (JHT), as a source of triterpenoids. JHT contains various triterpenoids and has shown favorable effects against dermatitis in both clinical and animal studies [18,19,20,21]. In this study, we selected 19 triterpenoids that originated from crude drugs of JHT component, and investigated whether these triterpenoids modulate the KLK5 protease activity.

## 2. Results

### 2.1. Triterpenoids Inhibit KLK5 Protease Activity

**Figure 1:**
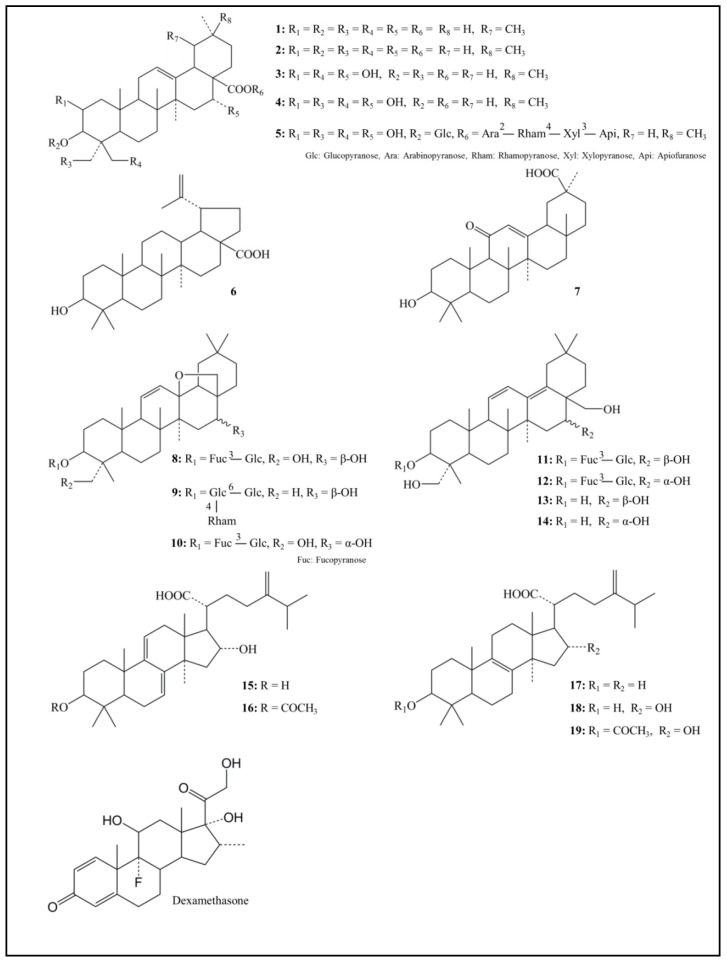
Chemical structure of 19 triterpenoids. **1**: Ursolic acid; **2**: Oleanolic acid; **3**: Polygalacic acid; **4**: Platycodigenin; **5**: Platycodin D; **6**: Betulinic acid; **7**: 18β-Glycyrrhetinic acid; **8**: Saikosaponin a; **9**: Saikosaponin c; **10**: Saikosaponin d; **11**: Saikosaponin b_1_; **12**: Saikosaponin b_2_; **13**: Saikogenin A; **14**: Saikogenin D; **15**: Dehydrotumulosic acid; **16**: Dehydropachymic acid; **17**: Eburicoic acid; **18**: Tumulosic acid; **19**: Pachymic acid.

The chemical structures of 19 triterpenoids, which are representative constituents of JHT, are shown in Figure 1. We first examined their potential to inhibit the KLK5 activity at 10 μM whereas only 18β-glycyrrhetinic acid was evaluated at 30 μM (Table 1).

**Figure 2:**
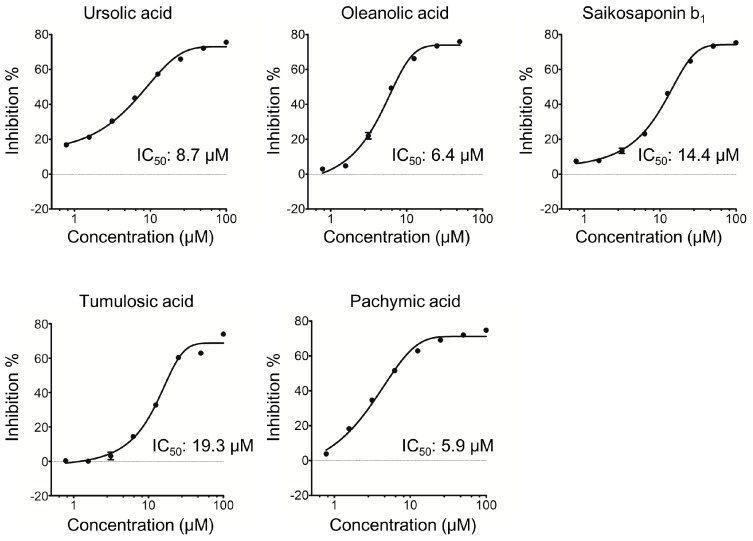
Dose-dependent anti-KLK5 activity of triterpenoids. All samples were evaluated at the described concentrations. The percentage of inhibition was calculated based on the formula: (1 − (A − B)/(C − B)) × 100, where A, test sample RFU; B, basal RFU without KLK5; and C, vehicle RFU. Data are presented as mean ± SEM of triplicate tests.

Ursolic acid, oleanolic acid, pachymic acid, dehydropachymic acid, tumulosic acid, saikosaponin b_1_, saikosaponin b_2_, and saikogenin A at 10 μM inhibited the hydrolysis of a synthetic substrate by purified KLK5, showing inhibition percentages of 54.6%, 51.6%, 56.2%, 33.9%, 36.0%, 39.6%, 21.1%, and 24.3%, respectively. The positive control agent, leupeptin hemisulfate, completely inhibited the KLK5 activity at 42.1 μM, while betulinic acid, polygalacic acid, and synthetic steroid dexamethasone did not influence the KLK5 activity. IC50 values for ursolic acid, oleanolic acid, pachymic acid, tumulosic acid and saikosaponin b_1_ were 8.7, 6.4, 5.9, 19.3, and 14.4 μM, respectively (Figure 2). Notably, the inhibitory effect of triterpenoids on KLK5 activity was dose-dependent.

We further investigated the selectivity of triterpenoids against the KLK5 protease activity using two representative triterpenoids: ursolic acid out of pentacyclic triterpenoids, and tumulosic acid out of tetracyclic triterpenoids. As shown in Table 2, ursolic acid inhibited KLK5 and trypsin activities; the IC_50_ values were 5.8 and 10.5 μM, respectively. In contrast, the IC_50_ values against KLK7 and chymotrypsin C activities were >100 μM. Ursolic acid exhibited a moderate inhibitory effect on KLK7 and chymotrypsin C at a maximal concentration of 100 μM (38.4% and 35.3%, respectively). Similarly, tumulosic acid showed IC_50_ values of 14.84 and 10.48 μM against KLK5 and trypsin proteases, while it exhibited a weak inhibitory effect on KLK7 and chymotrypsin C proteases (19.5% and 4.0%, respectively, at 100 μM).

### 2.2. Ursolic Acid and Tumulosic Acid Decreased LL-37 Production in Epidermal Keratinocytes

**Figure 3:**
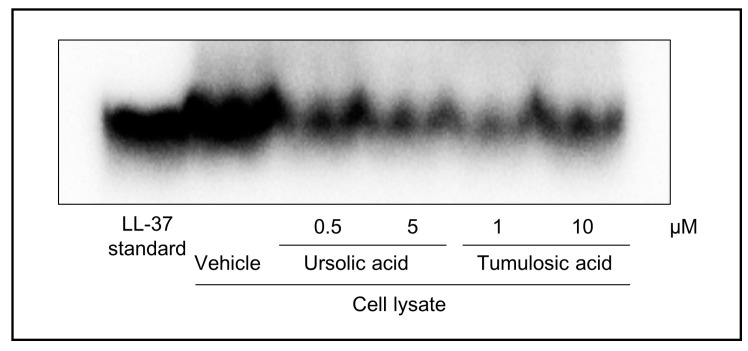
Decrease in LL-37 production in human keratinocytes treated with triterpenoids. Normal human epidermal keratinocytes (NHEKs) were treated with ursolic acid (0.5 μM and 5 μM) or tumulosic acid (1 μM and 10 μM) or vehicle for 23 h in the presence of 10 μM cycloheximide. LL-37 peptide in the lysate of cultured cells was detected using immunoprecipitation and Western blotting. Representative results are shown. Recombinant human LL-37 (0.2 ng) was loaded in the leftmost lane as a standard.

**Figure 4:**
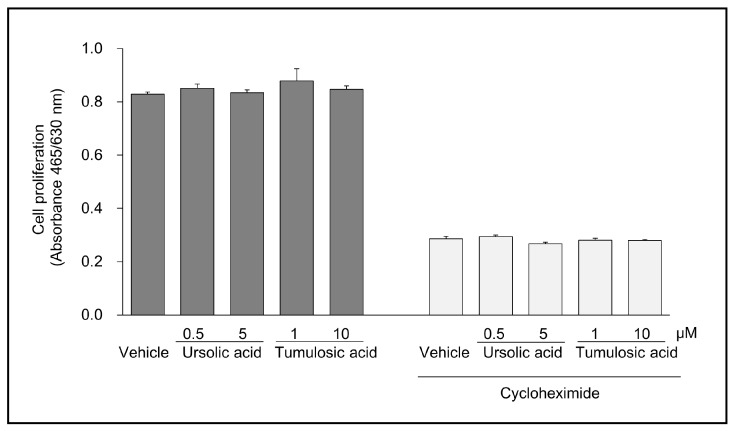
No cytotoxic effect of triterpenoids on cell proliferation. NHEKs were cultured with ursolic acid (0.5 μM and 5 μM) or tumulosic acid (1 μM and 10 μM) or vehicle in the presence or absence of 10 μM cycloheximide. After 24 h culture, cell proliferation activities were measured using an XTT-based cell viability assay kit. Optical density at 465 nm was measured by subtracting the reference absorbance at 630 nm. Data are presented as mean ± SEM of triplicate tests.

Because KLK5 is involved in the processing of CAMP in epidermal keratinocytes [5], we examined the effect of ursolic acid and tumulosic acid on CAMP LL-37 production in normal human epidermal keratinocytes (NHEKs). LL-37 in the lysates of NHEKs was detected using immunoprecipitation and Western blotting. As illustrated in Figure 3, the amount of LL-37 was decreased after the treatment with ursolic acid (0.5 and 5 μM) or tumulosic acid (1 and 10 μM) in the presence of 10 μM cycloheximide. No cytotoxic effect of both triterpenoids was observed at tested concentrations (Figure 4).

### 2.3. Pharmacokinetics of Triterpenoids in Rat after Oral Administration of JHT 

**Figure 5:**
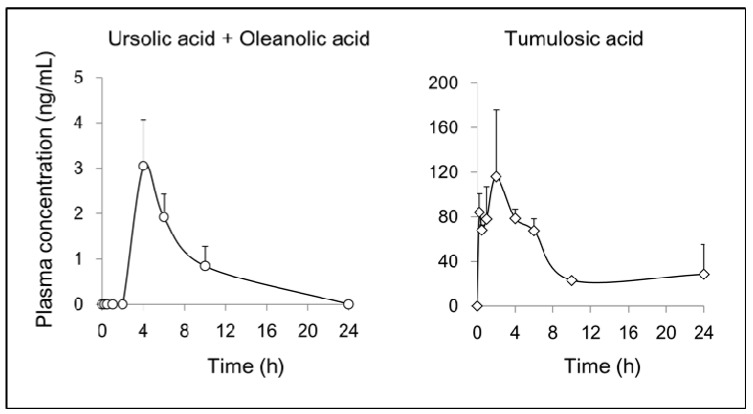
Plasma pharmacokinetic analysis of active triterpenoids of Jumihaidokuto. Plasma samples of rats were obtained at 0.25, 0.5, 1, 2, 4, 6, 10 and 24 h after single oral administration of Jumihaidokuto (JHT) (2 g/10 mL/kg, p.o.). Ursolic acid (and/or oleanolic acid) and tumulosic acid in the plasma were measured by LC-MS/MS. Each data point represented the mean ± SEM of triplicate results.

As shown in Figure 5, the *C*_max_ values of ursolic acid (and/or oleanolic acid) and tumulosic acid, were 3.1 ng/mL (4 h) and 116.1 ng/mL (2 h), respectively. Ursolic acid and oleanolic acid are structurally similar compounds. Both were inseparable in the plasma under reverse phase HPLC conditions in this study; therefore, the results represent combined values for both triterpenoids. The *C*_max_ value of pachymic acid was 2.1 ng/mL (6 h). Plasma concentrations of saikosaponin b_1_ were below the quantification limits (0.25 ng/mL).

## 3. Discussion

### 3.1. New Findings and Enzyme Selectivity

This is the first report to clarify the effects of naturally-occurring triterpenoids on human KLK5 protease. Ursolic acid and tumulosic acid suppressed the KLK5 activity as well as CAMP LL-37 production from NHEKs. Because KLK5 is involved in the processing of CAMP in epidermal keratinocytes [5], our findings suggest that ursolic acid and tumulosic acid regulate the proteolytic processing of KLK5 and modulate CAMP production in keratinocytes. Both ursolic acid and tumulosic acid inhibited KLK5 and trypsin activities, but not KLK7 and chymotrypsin C activities, which indicates that these triterpenoids are more selective against trypsin-like serine protease (KLK5) than against chymotrypsin-like serine protease (KLK7). Because the anti-protease activity was detected at a relatively low concentration of <100 µM and chymotrypsin-like serine protease (KLK7 and chymotrypsin C) activity was partially inhibited at 100 µM, triterpenoids may exhibit an anti-protease activity against other proteases at higher concentrations.

### 3.2. Structure–Activity Relationship

In the screening assay to examine the anti-KLK5 activity of 19 triterpenoids, some triterpenoids in both pentacyclic (ursolic acid, oleanolic acid, saikosaponin b_1_) and tetracyclic (pachymic acid, tumulosic acid) ring groups suppressed the KLK5 activity. Similar to our results, ursolic acid and oleanolic acid, but not 18β-glycyrrhetinic acid, were shown to inhibit the hydrolysis of a synthetic substrate by human leukocyte elastase, which belongs to the serine protease family [12,22]. In a study which examined the relationship between the structure and activity of triterpenoids, triterpenoids that inhibit the protease activity were found to have a hydroxy group and an appropriate side chain in the region of the molecule distal to the 3-hydroxy group [14]. Among the active triterpenoids that showed anti-KLK5 activity in our study, common critical molecular moieties in these triterpenoids were not identified. However, we are interested in the structure of betulinic acid, which is an analog of ursolic acid and oleanolic acid was inactive in the KLK5 assay as well as dexamethasone. Betulinic acid and dexamethasone do not have a double bond on ring C of the ring system, while ursolic acid and oleanolic acid do. This suggests that a double bond on ring C is necessary but is not sufficient for anti-KLK5 activity.

Fatty acid esterification or glucuronidation of the triterpenoid 3-hydroxy group increases the bioactivities [14,23]. Pachymic acid is a triterpenoid modified by acetoxylation at the 3-hydroxy group of tumulosic acid. In our study, pachymic acid showed a higher activity than did tumulosic acid (percentage of inhibition: 56.2% in pachymic acid vs. 36.0% in tumulosic acid), although the difference was not remarkable. Saikosaponin b_1_ and b_2_, which are glycosidated forms of saikogenin A and D, respectively, showed a higher activity than that showed by their aglycons (percentage of inhibition: 39.6% in saikosaponin b_1_ vs. 24.3% in saikogenin A and 21.1% in saikosaponin b_2_ vs. −1.5% in saikogenin D). These findings suggest that chemical modulation at the 3-hydroxy group in triterpenoids may contribute to an increase in the anti-KLK5 protease activity. Contrary to the effect of glycosylation in saikosaponin b_1_ and b_2_, the presence of an ether bond on ring D of the pentacyclic ring system may diminish the anti-KLK activity because of the lack of activity of saikosaponin a, c, and d.

### 3.3. Effect of Proteolytic Activity on LL-37 Production

CAMP is a family of polypeptides found in lysosomes of macrophages and polymorphonuclear leukocytes as well as in keratinocytes [24]. One of the peptides, LL-37, is known to be mainly produced by the KLK5-dependent proteolytic processing of cathelicidin in the epidermis [5]. Therefore, we evaluated the effect of active triterpenoids on the proteolytic processing of cathelicidin to produce LL-37 in a cell-culture system using NHEKs. We first measured the messenger RNA expression of cathelicidin and KLK5 and confirmed their expression in both undifferentiated and calcium-induced differentiated NHEKs (data not shown). The results of immunoprecipitation and Western blotting revealed that ursolic acid and tumulosic acid suppressed LL-37 production in the presence of protein synthesis inhibitor, cycloheximide. Further, no cytotoxic effect of these acids was observed at concentrations up to 10 μM, as shown by the results of a cell viability assay based on metabolic activity. These findings indicate that ursolic acid and tumulosic acid suppressed LL-37 production probably via the inhibition of KLK5 activity and the proteolytic conversion of CAMP to LL-37.

### 3.4. Potential of Triterpenoids as Internal Drugs

Jumihaidokuto (JHT) is an extract mixture of the following ten crude components: Platycodon Root, Bupleurum Root, Cnidium Rhizome, Poria sclerotium, Quercus Bark, Aralia Rhizome, Saposhnikovia Root and Rhizome, Glycyrrhiza, Schizonepeta Spike, and Ginger. Fourteen pentacyclic triterpenoids used in this study supposedly originated from Schizonepeta Spike, Bupleurum Root, and Platycodon Root, whereas five tetracyclic triterpenoids originated from Poria sclerotium. Schizonepeta Spike and Poria sclerotium contain ursolic acid and tumulosic acid, respectively [25,26]. These have been shown to be beneficial in animal models of skin dermatoses [27,28,29,30]. We conducted a plasma pharmacokinetic study of triterpenoids wherein JHT was orally administrated to rats at 2 g/kg. As a result of time course measurement up to 24 h after a single oral administration of JHT, ursolic acid (and/or oleanolic acid), tumulosic acid, and pachymic acid were detected in the plasma as shown in Figure 5; pharmacokinetics of those triterpenoids were previously reported by us and others [31,32,33].

In contrast, saikosaponin b_1_ was not detected in the plasma in this study (data not shown). The higher absorbance of tumulosic acid than of other triterpenoids is a notable finding; the *C*_max_ of tumulosic acid was 116.1 ng/mL (0.238 μM). High concentration of tumulosic acid in blood was reported after the oral administration of crude drugs containing Poria sclerotium [33,34,35]. Further pharmacokinetic studies of bioactive triterpenoids are required to examine plasma absorption and distribution in skin tissue; the results will help us understand their potential use as internal agents.

JHT has been widely used for the treatment of skin symptoms such as reddening, swelling, sharp pain, and a sense of burning, as well as for the treatment of skin diseases including acute and/or purulent skin diseases, urticaria, eczema, and athlete's foot. JHT is effective in patients with inflammatory acne and palmoplantar pustulosis [18,19]. The anti-KLK5 protease activity of triterpenoids obtained from crude components of JHT has not been reported. Our study reveals a potential mechanism by which JHT helps in the maintenance of the skin barrier function, i.e., via blood-absorbed triterpenoids with the anti-KLK5 activity. Because there is no drug to regulate KLK5, our findings pave the way for future studies to investigate the potential use of triterpenoids as an anti-KLK5 drug and as a therapeutic drug against refractory skin diseases such as rosacea and atopic dermatitis.

### 3.5. Summary: Possible Effects of Triterpenoids on Dermatoses

There is a growing body of studies on the effects of the oral or topical administration of triterpenoids, including ursolic acid, oleanolic acid and pachymic acid in animal models of atopic dermatitis, allergic contact dermatitis, inflammation, and skin tumor promotion by 12-*O*-tetradecanoylphorbol-13-acetate [16,17]. Furthermore, ursolic acid and oleanolic acid have been shown to improve the recovery of the skin barrier function in a tape-stripping model [15]. Ursolic acid is used as an additive in cosmetic products. Triterpenoids surely act on multiple target molecules such as PPAR-alpha and kinase enzymes; however, it is possible that the above effects of triterpenoids reflect their anti-KLK5 activity.

Although the present study could not clarify an evident structure–activity relationship of triterpenoids, well-designed further studies on intermolecular interactions will promote the development of a new drug of KLK5 inhibitors. Our finding has led to the future possibility and necessity to conduct further research on triterpenoids as a therapeutic drug on refractory skin diseases such as rosacea and atopic dermatitis.

## 4. Materials and Methods

### 4.1. Tested Triterpenoids and Reference Regents

Ursolic acid, oleanolic acid, saikosaponin a, saikosaponin b_2_, saikosaponin c, saikosaponin d, and platycodin D were purchased from Wako Pure Chemical Industries (Osaka, Japan). Platycodigenin, polygalacic acid, dehydropachymic acid, dehydrotumulosic acid, and eburicoic acid were purchased from Wuhan ChemFaces Biochemical Co., Ltd. (Wuhan, China). Tumulosic acid was purchased from Carbosynth (Compton, Berkshire, UK). 18β-glycyrrhetinic acid, saikosaponin b_1_, saikogenin A, saikogenin D, pachymic acid and betulinic acid with sufficiently high purities to be evaluated for biological tests were supplied by Tsumura & Co. (Tokyo, Japan). Dexamethasone (Wako Pure Chemical Industries), leupeptin hemisulfate salt and chymostatin (Sigma-Aldrich, St. Louis, MO, USA) were purchased for use as reference regents.

### 4.2. Measurements of Each Enzymatic Activity

KLK5, KLK7, trypsin, and chymotrypsin C activities were evaluated according to the following methods supplied by R & D Systems Inc. (Minneapolis, MN, USA). The enzyme activity-dependent increase in the relative fluorescent unit (RFU) was measured, and the percentage of inhibition was calculated based on the formula: (1 − (A − B)/(C − B)) × 100, where A = RFU of test samples with enzymes, B = basal RFU without enzymes, and C = RFU of vehicle controls with enzymes.

To measure the KLK5 activity, enzymatic reaction was performed at room temperature in 100 mM NaH_2_PO_4_ buffer (pH 8.0) containing 0.25 μg/mL recombinant human KLK5 (R & D Systems Inc.), 100 μM of Boc-V-P-R-AMC Fluorogenic Peptide Substrate (R & D Systems Inc.), and 1.1% DMSO at final concentrations. KLK5 (final 8.1 nM) was preincubated with test samples for 5 min, followed by the addition of peptide substrate. After incubating for 5 min, RFU was measured at Ex 380 nm/Em 460 nm. Leupeptin hemisulfate (42.1 μM) was used as a positive control.

To measure the KLK7 activity, recombinant human pro-KLK7 (R & D Systems Inc.) was activated by bacterial thermolysin at 37 °C for 2 h just before the enzyme assay. Thereafter, enzymatic reaction was performed at room temperature in 50 mM Tris, 150 mM NaCl buffer (pH 8.5) containing 1 μg/mL activated-KLK7, 10 μM Mca-R-P-K-P-V-E-Nval-W-R-K (Dnp)-NH_2_ Fluorogenic Peptide Substrate II (R & D Systems Inc.), 150 mM NaCl, and 1.1% DMSO at final concentrations. Activated KLK7 (final 38.5 nM) was preincubated with test samples for 5 min, followed by the addition of peptide substrate. After incubating for 60 min, RFU was measured at Ex 320 nm/Em 405 nm. Chymostatin (10 μM) was used as a positive control.

To measure the trypsin activity, enzymatic reaction was performed at room temperature in 100 mM NaH_2_PO_4_ buffer (pH 8.0) containing 0.25 μg/mL recombinant human trypsin (Wako Pure Chemical Industries), 100 μM of Boc-V-P-R-AMC Fluorogenic Peptide Substrate, and 1.1% DMSO at final concentrations. KLK5 was preincubated with test samples for 5 min, followed by the addition of peptide substrate. After incubating for 1 min, RFU was measured at Ex 380 nm/Em 460 nm. Leupeptin hemisulfate (42.1 μM) was used as a positive control.

To measure the chymotrypsin C activity, recombinant human pro-chymotrypsin C (R & D Systems Inc.) was activated by trypsin at 37 °C for 1 h just before the enzyme assay. Thereafter, the enzymatic reaction was performed at room temperature in 25 mM Tris, 0.5 mM CaCl_2_ buffer (pH 8.0) containing 1 μg/mL activated chymotrypsin C, 10 μM Suc-A-A-P-F-AMC (Bachem AG, Bubendorf, Switzerland) used as a fluorogenic substrate, and 1.1% DMSO at final concentrations. Activated chymotrypsin C (final 34.7 nM) was preincubated with test samples for 5 min, followed by the addition of peptide substrate. After incubating for 60 min, RFU was measured at Ex 380 nm/Em 460 nm. Chymostatin (10 μM) was used as a positive control.

### 4.3. Detection of LL-37 by Immunoprecipitation

NHEKs were purchased from Takara Bio Inc. (Shiga, Japan) and cultured in keratinocyte growth medium 2 (PromoCell GmbH, Heidelberg, Germany) supplemented with 4 μL/mL bovine pituitary extract, 0.125 ng/mL epidermal growth factor, 5 μg/mL insulin, 0.33 μg/mL hydrocortisone, 0.39 μg/mL epinephrine, 10 μg/mL transferrin, and 0.06 mM CaCl_2_ in a humidified atmosphere of 5% CO_2_ at 37 °C. NHEKs (6 × 10^5^ cells/4 mL) were seeded in 60-mm tissue culture dishes and treated with 10 μM cycloheximide (Wako, Osaka, Japan) to inhibit protein synthesis. After 1 h, ursolic acid (0.5 μM and 5 μM) or tumulosic acid (1 μM and 10 μM) was added to the culture and incubated for an additional 23 h. NHEKs were rinsed with cold phosphate buffered saline (-) and lysed with 0.5 mL lysis buffer (50 mM Tris-HCl, 150 mM NaCl, 0.02% sodium azide, 1% triton X-100). Cell lysates were centrifuged at 10,000 *g* for 10 min, and supernatants were collected. Protein concentration was measured using the BCA protein assay kit (Thermo Fisher Science, Waltham, MA, USA).

Immunoprecipitation was performed using an immunoprecipitation kit of dynabeads protein G (Thermo Fisher Science) according to the manufacturers’ protocol. Target antigen was eluted by 2× Laemmli sample buffer (Bio-Rad, Hercules, CA, USA) containing 10% β-mercaptoethanol and denatured at 95 °C for 5 min. To detect LL37, proteins separated by SDS-PAGE were transferred to PVDF membrane (GE healthcare, Little Chalfont, UK). The membrane was blocked using 0.3% skim milk TBS-T (20 mM Tris-HCl, 137 mM NaCl, 0.05% Tween 20, pH 7.6) at room temperature for 1 h and incubated overnight at 4 °C in 0.3% skim milk containing 400 ng/mL anti-LL-37 rabbit polyclonal antibody (LifeSpan BioScience, Seattle, WA, USA). Subsequently, the membrane was incubated with goat anti-rabbit IgG peroxidase conjugated antibody (1:2000 in 0.3% skim milk TBS-T). A standard LL37 peptide (Peptide Institute, Osaka, Japan) was loaded at 0.2 ng/lane as a positive control. Signals were developed using the EzWestLumi plus (ATTO, Tokyo, Japan) for 5 min and imaged using the ChemiDoc XRS (Bio-Rad).

### 4.4. Cell Growth Test

NHEKs were plated in 96-well flat bottom microtiter plates at 2 × 10^4^ cells/well in the medium supplemented with the same additives described above and were allowed to settle overnight. Subsequently, culture fluids were replaced with fresh medium containing test sample or test sample and 10 μM cycloheximide. After 24 h culture, cell growth activities were measured using an XTT-based Cell Proliferation Kit (Biological Industries, Beit Haemek, Israel) according to the manufacturer’s instructions. Optical density at 465 nm was measured by subtracting the reference absorbance at 630 nm. The optical density of medium-only wells was in the range of 0.07–0.10.

### 4.5. Pharmacokinetic Analysis of Triterpenoids in Rats

Male Sprague–Dawley rats were purchased from Charles River Laboratories (Yokohama, Japan). The plasma pharmacokinetic study was performed according to the “Guidelines for the Care and Use of Laboratory Animals” of Tsumura & Co. Ethical approval for experimental procedures was obtained from the Laboratory Animal Committee of Tsumura & Co. Jumihaidokuto (JHT, Lot No. 332031900) was supplied by Tsumura & Co. (Tokyo, Japan) in the form of a powdered extract. JHT prepared in distilled water was orally administered to 16-h fasted rats at 2 g/10 mL/kg (*n* = 3). Blood samples were obtained from the abdominal inferior vena cava using a heparinized syringe at 0.25, 0.5, 1, 2, 4, 6, 10, and 24 h after the administration of JHT. Plasma was obtained by centrifugation at 1700 *g* for 15 min at 4 °C and stored at −80 °C until further processing.

Ursolic acid (and/or oleanolic acid), saikosaponin b_1_, pachymic acid, and tumulosic acid were measured in the present study. Twenty-five microliters of methanol, an equal volume of atropine (2 ng/mL; Wako Pure Chemical Industries) as internal standard, and 750 µL of methanol were added to 200 µL of plasma samples, followed by mixing and centrifugation (7000× *g*, 5 min). For the preparation of the calibration curve, the same volumes of various concentrations of working solution were used instead of methanol. The supernatant was dried at 40 °C under a stream of nitrogen gas. The dried residue was dissolved in 50–60 μL of the initial mobile phase used for liquid chromatography–mass spectrometry with a tandem mass spectrometry system (LC-MS/MS; API4000 or Triple Quad 6500; AB Sciex, Tokyo, Japan) and then a 10- or 20-μL portion was injected into the LC-MS/MS system for the quantification of triterpenoids.

### 4.6. Statistical Analysis

All data are presented as mean ± standard error of the mean (SEM). Statistical significance was evaluated using Dunnett’s multiple comparisons. A *p*-value of <0.05 was considered significant.

## Figures and Tables

**Table 1:** Random assay to identify triterpenoids with anti-KLK5 activity.

Structure	Compound	Anti-KLK5 Activity % Inhibition
Pentacyclic triterpenoid		
	1 Ursolic acid	54.6 ± 1.3
	2 Oleanolic acid	51.6 ± 1.8
	3 Polygalacic acid	5.5 ± 2.3
	4 Platycodigenin	8.8 ± 2.0
	5 Platycodin D	−6.0 ± 2.9
	6 Betulinic acid	5.5 ± 2.5
	7 18β-Glycyrrhetinic acid	16.0 ± 1.2
	8 Saikosaponin a	4.3 ± 0.7
	9 Saikosaponin c	4.1 ± 1.6
	10 Saikosaponin d	−10.4 ± 3.5
	11 Saikosaponin b_1_	39.6 ± 1.6
	12 Saikosaponin b_2_	21.1 ± 1.4
	13 Saikogenin A	24.3 ± 2.6
	14 Saikogenin D	−1.5 ± 3.6
Tetracyclic triterpenoid		
	15 Dehydrotumulosic acid	−46.2 ± 2.7
	16 Dehydropachymic acid	33.9 ± 3.7
	17 Eburicoic acid	−51.0 ± 3.0
	18 Tumulosic acid	36.0 ± 1.9
	19 Pachymic acid	56.2 ± 1.5
Reference		
	Dexamethasone	−1.8 ± 2.8
	Leupeptin hemisulfate	96.9 ± 0.1

Recombinant human kallikrein 5 (KLK5, 8.1 nM) was mixed with 10 μM triterpenoid and Boc-Val-Pro-Arg-AMC fluorogenic peptide (100 μM), whereas only 18β-glycyrrhetinic acid was evaluated at 30 μM. After incubating for 5 min, the relative fluorescent unit (RFU) was measured at Ex 380 nm/Em 460 nm. A serine protease inhibitor, leupeptin hemisulfate (42.1 μM), was used as positive control. Triterpenoids showing an activity of more than 20% inhibition versus control were statistically significant at each concentration by Dunnett’s test. Data are presented as the mean ± SEM of triplicate tests. The percentage of inhibition was calculated using the formula: (1 − (A − B)/(C − B)) × 100, where A, test sample RFU; B, basal RFU without KLK5; and C, vehicle RFU.

**Table 2:** Comparative activities of ursolic acid and tumulosic acid against serine proteases.

	Ursolic Acid		Tumulosic Acid	
	IC_50_ (μM)	Inhibition % at 100 μM	IC_50_ (μM)	Inhibition % at 100 μM
Kallikrein 5	5.8	74.5 ± 0.2	14.8	58.7 ± 0.2
Kallikrein 7	>100	38.4 ± 1.6	>100	19.5 ± 1.5
Trypsin	14.8	76.9 ± 3.5	45.3	73.2 ± 1.6
Chymotrypsin C	>100	35.3 ± 2.9	>100	4.1 ± 5.2

Ursolic acid and tumulosic acid were evaluated at 0.78, 1.56, 3.13, 6.25, 12.5, 50, and 100 μM in enzymatic assays for kallikrein 5, kallikrein 7, trypsin, and chymotrypsin C. Each enzyme activity-dependent increase in the relative fluorescent unit (RFU) was measured, and the percentage of inhibition was calculated based on the formula: (1 − (A − B)/(C − B)) × 100, where A, test sample RFU; B, basal RFU without KLK5; and C, vehicle RFU. The 50% inhibitory concentration (IC_50_) values were calculated in each assay by linear interpolation. Protease activities with ursolic acid or tumulosic acid at 100 µM are also presented.

## References

[1] Brattsand M., Stefansson K., Lundh C., Haasum Y., Egelrud T. (2005). A proteolytic cascade of kallikreins in the stratum corneum. J. Investig. Dermatol..

[2] Caubet C., Jonca N., Brattsand M., Guerrin M., Bernard D., Schmidt R., Egelrud T., Simon M., Serre G. (2004). Degradation of corneodesmosome proteins by two serine proteases of the kallikrein family, SCTE/KLK5/hK5 and SCCE/KLK7/hK7. J. Investig. Dermatol..

[3] Schechter N.M., Choi E.J., Wang Z.M., Hanakawa Y., Stanley J.R., Kang Y., Clayman G.L., Jayakumar A. (2005). Inhibition of human kallikreins 5 and 7 by the serine protease inhibitor lympho-epithelial Kazal-type inhibitor (LEKTI). Biol. Chem..

[4] Furio L., Pampalakis G., Michael I.P., Nagy A., Sotiropoulou G., Hovnanian A. (2015). KLK5 inactivation reverses cutaneous hallmarks of netherton syndrome. PLoS Genet..

[5] Yamasaki K., Schauber J., Coda A., Lin H., Dorschner R.A., Schechter N.M., Bonnart C., Descargues P., Hovnanian A., Gallo R.L. (2006). Kallikrein-mediated proteolysis regulates the antimicrobial effects of cathelicidins in skin. FASEB J..

[6] Yamasaki K., Di Nardo A., Bardan A., Murakami M., Ohtake T., Coda A., Dorschner R.A., Bonnart C., Descargues P., Hovnanian A. (2007). Increased serine protease activity and cathelicidin promotes skin inflammation in rosacea. Nat. Med..

[7] Yamasaki K., Kanada K., Macleod D.T., Borkowski A.W., Morizane S., Nakatsuji T., Cogen A.L., Gallo R.L. (2011). TLR2 expression is increased in rosacea and stimulates enhanced serine protease production by keratinocytes. J. Investig. Dermatol..

[8] Yamasaki K., Gallo R.L. (2011). Rosacea as a disease of cathelicidins and skin innate immunity. J. Investig. Dermatol. Symp. Proc..

[9] Komatsu N., Saijoh K., Kuk C., Liu A.C., Khan S., Shirasaki F., Takehara K., Diamandis E.P. (2007). Human tissue kallikrein expression in the stratum corneum and serum of atopic dermatitis patients. Exp. Dermatol..

[10] Briot A., Deraison C., Lacroix M., Bonnart C., Robin A., Besson C., Dubus P., Hovnanian A. (2009). Kallikrein 5 induces atopic dermatitis-like lesions through PAR2-mediated thymic stromal lymphopoietin expression in Netherton syndrome. J. Exp. Med..

[11] Goettig P., Magdolen V., Brandstetter H. (2010). Natural and synthetic inhibitors of kallikrein-related peptidases (KLKs). Biochimie.

[12] Safayhi H., Rall B., Sailer E.R., Ammon H.P. (1997). Inhibition by boswellic acids of human leukocyte elastase. J. Pharmacol. Exp. Ther..

[13] Feng L., Liu X., Zhu W., Guo F., Wu Y., Wang R., Chen K., Huang C., Li Y. (2013). Inhibition of human neutrophil elastase by pentacyclic triterpenes. PLoS ONE.

[14] Rajic A., Akihisa T., Ukiya M., Yasukawa K., Sandeman R.M., Chandler D.S., Polya G.M. (2001). Inhibition of trypsin and chymotrypsin by anti-inflammatory triterpenoids from Compositae flowers. Planta Med..

[15] Lim S.W., Hong S.P., Jeong S.W., Kim B., Bak H., Ryoo H.C., Lee S.H., Ahn S.K. (2007). Simultaneous effect of ursolic acid and oleanolic acid on epidermal permeability barrier function and epidermal keratinocyte differentiation via peroxisome proliferator-activated receptor-alpha. J. Dermatol..

[16] Cuellar M.J., Giner R.M., Recio M.C., Just M.J., Manez S., Rios J.L. (1997). Effect of the basidiomycete *Poria cocos* on experimental dermatitis and other inflammatory conditions. Chem. Pharm. Bull..

[17] Choi J.K., Oh H.M., Lee S., Park J.W., Khang D., Lee S.W., Lee W.S., Rho M.C., Kim S.H. (2013). Oleanolic acid acetate inhibits atopic dermatitis and allergic contact dermatitis in a murine model. Toxicol. Appl. Pharmacol..

[18] Higaki S., Toyomoto T., Morohashi M. (2002). Seijo-bofu-to, Jumi-haidoku-to and Toki-shakuyaku-san suppress rashes and incidental symptoms in acne patients. Drugs Exp. Clin. Res..

[19] Mizawa M., Makino T., Inami C., Shimizu T. (2016). Jumihaidokuto (Shi-Wei-Ba-Du-Tang), a kampo formula, decreases the disease activity of palmoplantar pustulosis. Dermatol. Res. Pract..

[20] Sekiguchi K., Koseki J., Tsuchiya K., Matsubara Y., Iizuka S., Imamura S., Matsumoto T., Watanabe J., Kaneko A., Aiba S. (2015). Suppression of *Propionibacterium acnes*-induced dermatitis by a traditional Japanese medicine, jumihaidokuto, modifying macrophage functions. Evid. Based Complent. Alternat. Med..

[21] Nose M., Sakushima J., Harada D., Ogihara Y. (1999). Comparison of immunopharmacological actions of 8 kinds of kampo-hozais clinically used in atopic dermatitis on delayed-type hypersensitivity in mice. Biol. Pharm. Bull..

[22] Ying Q.L., Rinehart A.R., Simon S.R., Cheronis J.C. (1991). Inhibition of human leucocyte elastase by ursolic acid. Evidence for a binding site for pentacyclic triterpenes. Biochem. J..

[23] Yoshikawa M., Matsuda H. (2000). Antidiabetogenic activity of oleanolic acid glycosides from medicinal foodstuffs. Biofactors.

[24] Yamasaki K., Gallo R.L. (2008). Antimicrobial peptides in human skin disease. Eur. J. Dermatol..

[25] Zhang L., Feng Y., Ding A. (2001). The research on the chemical components of *Schizonepeta tenuifolia* Briq. J. Chin. Med. Mater..

[26] Rios J.L. (2011). Chemical constituents and pharmacological properties of *Poria cocos*. Planta Med..

[27] Choi Y.Y., Kim M.H., Kim J.H., Jung H.S., Sohn Y., Choi Y.J., Hwang M.K., Kim S.H., Kim J., Yang W.M. (2013). *Schizonepeta tenuifolia* inhibits the development of atopic dermatitis in mice. Phytother. Res..

[28] Tohda C., Kakihara Y., Komatsu K., Kuraishi Y. (2000). Inhibitory effects of methanol extracts of herbal medicines on substance P-induced itch-scratch response. Biol. Pharm. Bull..

[29] Fung D., Lau C.B. (2002). *Schizonepeta tenuifolia*: Chemistry, pharmacology, and clinical applications. J. Clin. Pharmacol..

[30] Nukaya H., Yamashiro H., Fukazawa H., Ishida H., Tsuji K. (1996). Isolation of inhibitors of TPA-induced mouse ear edema from Hoelen, *Poria cocos*. Chem. Pharm. Bull..

[31] Chen Q., Luo S., Zhang Y., Chen Z. (2011). Development of a liquid chromatography-mass spectrometry method for the determination of ursolic acid in rat plasma and tissue: Application to the pharmacokinetic and tissue distribution study. Anal. Bioanal. Chem..

[32] Jeong D.W., Kim Y.H., Kim H.H., Ji H.Y., Yoo S.D., Choi W.R., Lee S.M., Han C.K., Lee H.S. (2007). Dose-linear pharmacokinetics of oleanolic acid after intravenous and oral administration in rats. Biopharm. Drug Dispos..

[33] Matsubara Y., Matsumoto T., Sekiguchi K., Koseki J., Kaneko A., Yamaguchi T., Kurihara Y., Kobayashi H. (2017). Oral administration of the Japanese traditional medicine keishibukuryogan-ka-yokuinin decreases reactive oxygen metabolites in rat plasma: Identification of chemical constituents contributing to antioxidant activity. Molecules.

[34] Lv C., Li Q., Zhang Y., Sui Z., He B., Xu H., Yin Y., Chen X., Bi K. (2013). A UFLC-MS/MS method with a switching ionization mode for simultaneous quantitation of polygalaxanthone III, four ginsenosides and tumulosic acid in rat plasma: Application to a comparative pharmacokinetic study in normal and Alzheimer’s disease rats. J. Mass Spectrom..

[35] Xiao F., Li Q., Liang K., Zhao L., He B., Ji W., Chen X., Wang Z., Bi K., Jia Y. (2012). Comparative pharmacokinetics of three triterpene acids in rat plasma after oral administration of Poria extract and its formulated herbal preparation: GuiZhi-FuLing capsule. Fitoterapia.

